# Human saliva as route of inter-human infection for mouse mammary tumor virus

**DOI:** 10.18632/oncotarget.4567

**Published:** 2015-07-15

**Authors:** Chiara Maria Mazzanti, Francesca Lessi, Ivana Armogida, Katia Zavaglia, Sara Franceschi, Mohammad Al Hamad, Manuela Roncella, Matteo Ghilli, Antonio Boldrini, Paolo Aretini, Giovanni Fanelli, Ivo Marchetti, Cristian Scatena, Jacob Hochman, Antonio Giuseppe Naccarato, Generoso Bevilacqua

**Affiliations:** ^1^ FPS – The Pisa Science Foundation, Pisa, Italy; ^2^ Department of Pathology, Pisa University Hospital, Pisa, Italy; ^3^ Breast Center, Department of Breast Surgery, Pisa University Hospital, Pisa, Italy; ^4^ Department of Neonatology, Pisa University Hospital, Pisa, Italy; ^5^ Department of Cell and Developmental Biology, Institute of Life Sciences, The Hebrew University of Jerusalem, Jerusalem, Israel; ^6^ Laboratory of Pathology, “San Rossore” Hospital, Pisa, Italy; ^7^ Current address: Illumina Cambridge Ltd., Cambridge, UK; ^8^ Current address: Department of Pathology, University of Dammam, Dammam, KSA

**Keywords:** Pathology Section, mouse mammary tumor virus (MMTV), breast cancer, breast cancer etiology, saliva

## Abstract

Etiology of human breast cancer is unknown, whereas the Mouse Mammary Tumor Virus (MMTV) is recognized as the etiologic agent of mouse mammary carcinoma. Moreover, this experimental model contributed substantially to our understanding of many biological aspects of the human disease. Several data strongly suggest a causative role of MMTV in humans, such as the presence of viral sequences in a high percentage of infiltrating breast carcinoma and in its preinvasive lesions, the production of viral particles in primary cultures of breast cancer, the ability of the virus to infect cells in culture. This paper demonstrates that MMTV is present in human saliva and salivary glands. MMTV presence was investigated by fluorescent PCR, RT-PCR, FISH, immunohistochemistry, and whole transcriptome analysis. Saliva was obtained from newborns, children, adults, and breast cancer patients. The saliva of newborns is MMTV-free, whereas MMTV is present in saliva of children (26.66%), healthy adults (10.60%), and breast cancer patients (57.14% as DNA and 33.9% as RNA). MMTV is also present in 8.10% of salivary glands. RNA-seq analysis performed on saliva of a breast cancer patient demonstrates a high expression of MMTV RNA in comparison to negative controls. The possibility of a contamination by murine DNA was excluded by murine mtDNA and IAP LTR PCR. These findings confirm the presence of MMTV in humans, strongly suggest saliva as route in inter-human infection, and support the hypothesis of a viral origin for human breast carcinoma.

## INTRODUCTION

The etiology of human breast carcinoma is unknown, with the exception of hereditary tumors, where inherited gene mutations are able to confer a high risk of developing cancer, and of radiation induced tumors. Estrogens are well known as relevant pathogenetic factors, but a transforming role could never be shown for them [1].

On the other hand, the Mouse Mammary Tumor Virus (MMTV) has been demonstrated as the causative agent of tumors of the mammary gland in mouse [2, 3]. In this context, attention needs to be focused on the fact that the MMTV model of breast carcinoma taught us much of what is known about the pathogenesis of the human sporadic breast carcinoma (HSBC) [4], in particular the concepts of cancer progression, preinvasive lesions, and promotional role of estrogens.

The quest for a possible viral etiology of HSBC was unsuccessful until 1995, when the laboratory of Beatriz Pogo selected a 660-bp region of MMTV with a nucleotide sequence identity of only 16% to human endogenous retroviral element K10, which is highly similar to MMTV, and 90% to 98% identical to the MMTV envelope (env) gene [5]. Moreover, the same authors, by using MMTV-specific primers located in the 660-bp region, identified a MMTV env gene–like exogenous sequence (MMTVels) in 38–40% of infiltrating HSBC.

These data were confirmed by several different groups, but negative results were also published. In 2006 we were able to design a rigorous methodological approach able to overcome any possible difficulty, and to definitively demonstrate that MMTV sequences are present in infiltrative HSBC in a percentage between 30% and 40% [6].

In the meantime, the isolation of viral particles from primary cultures of human breast cancer cells [7], the demonstration that MMTV can infect human cells [8], and the discovery of the viral origin of cervical carcinoma by HPV [9] gave more attention to the possibility that a virus could be involved in the initiation of HSBC.

In 2011 [10], we showed the presence of MMTVels in the different phases of HSBC progression, from normal gland tissue to infiltrative carcinoma via atypical epithelial hyperplasia and ductal carcinoma in situ (DCIS). At the same time, by real-time quantitative PCR we showed that a different viral load correlated with the stage of tumor progression. Moreover, by chromogenic in situ hybridization (CISH) we obtained very clear signals indicating the nuclear presence of MMTVels.

Recently, two interesting papers showed the presence of MMTV in human milk and in human milk cells [11, 12].

Surprisingly, in a recent study searching for known and novel viruses in human tumors, massive sequencing was not able to individuate MMTV [13], but a careful reading of the complete analysis (http://larssonlab.org/tcga-viruses/report_BRCA.php) shows the presence of a low MMTV expression in some breast cancer samples.

All considered, the amount of information available today strongly supports the hypothesis that MMTV plays a causative role in HSBC. However, how the virus could infect humans still remains an open question.

Once more, the murine MMTV model was of help: in mice MMTV is expressed also in salivary glands [14]. As saliva is a common route of spreading of viral infections, we decided to investigate human salivary glands and saliva and we were successful in demonstrating in both of them the presence of MMTV.

## RESULTS

### MMTV sequences are present in human saliva, with the exception of newborns

Saliva samples were collected at the Pisa University Hospital from a total of 235 consecutive individuals, grouped as follows: 56 breast cancer female patients, 132 healthy adult blood donors of both genders, 30 pediatric patients of both genders, 17 newborns of both genders (Table 1). Samples were collected anonymously according to the rules of the Ethics Committee of the Pisa University Hospital.

**Table 1:** Presence of MMTVels in human saliva and salivary glands

MMTVels in human saliva		DNA		RNA		DNA and RNA					
	total cases	positive cases		positive cases		DNA +/RNA+		DNA +/RNA −		DNA −/RNA +	
		n	%	n	%	n	%	n	%	n	%
a) newborns	17	0	0								
b) children	30	8	26.66								
c) adult healthy donors	132	14	10.60								
d) breast cancer patients	56	32	57.14	19	33.9	13	23.21	19	33.92	6	10.71
a+b+c+d	235	54	22.97								
b+c+d	218	54	24.77								
c+d	188	46	24.46								
**MMTVels in human salivary glands**		**DNA**									
	**total cases**	**positive cases**		**negative cases**							
		**n**	**%**	**n**	**%**						
	37	3	8.10	34	91.9						

a) **Saliva:** MMTV DNA is absent in newborns; in children it is present in a percentage more than double than in healthy adults; in breast cancer patients the percentage of positivity is five time that in healthy adults. MMTV RNA is present in one third of breast cancer patients. Discrepancy in percentage of positivity between MMTV DNA and RNA can be consequence of a different MMTV status, in active replication or in latency. b) **Salivary glands:** MMTVels are present in a percentage (8.10%) very similar to that of healthy adults with positive saliva (10.60%)

MMTV DNA was present in 26.66% (8/30 cases) of children, with 25% of females (5/20), and 30% of males (3/10). In adults, 10.6% resulted positive (14/132), with 15% of females (12/78) and 3.7% of males (2/54).

For what concerns breast cancer patients, MMTV DNA was present in 57.14% of cases (32/56), whereas MMTV RNA was present in 33.9% of cases (19/56). A simultaneous presence of viral DNA and RNA occurred in 23.21% of cases (13/56). On the other hand, a combination DNA positive/RNA negative was found in 33.92% of cases (19/56) and a combination DNA negative/RNA positive was present in 10.71% of cases (6/56).

The fact that saliva of newborns was free of MMTV DNA in all cases needs to be stressed.

### A whole transcriptome analysis of MMTV confirms the presence of viral RNA in saliva

**Figure 1:**
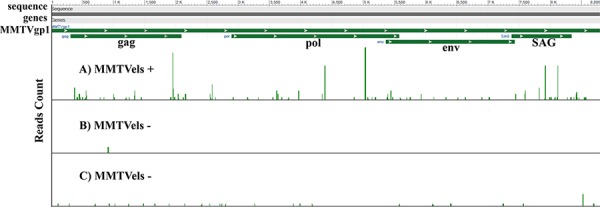
Whole transcriptome analysis of human saliva Reads count mapped to MMTV genome. **A.** MMTVels positive sample for a breast cancer patient. **B** and **C.** MMTVels negative human breast tissues used as negative control. A strong difference between A and B/C is easily evident.

RNA-seq analysis performed on a DNA MMTVels positive saliva from a breast cancer patient and on two DNA MMTVels negative normal breast tissues demonstrated the presence of RNA MMTV in the saliva with an expression value of 0.26 FPKM, whereas it was of 0.00 FPKM and 0.04 FPKM in the two breast tissue samples used as negative controls (Figure 1).

### FISH shows a cytoplasmic positivity in saliva cells

**Figure 2:**
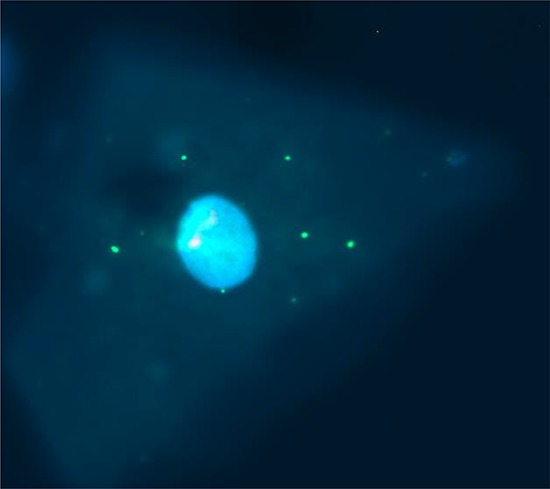
FISH analysis of MMTVels in human saliva MMTV signals in the cytoplasm of a possible epithelial exfoliated cell. In the nucleus a positive p53 signal is evident as control.

FISH analysis on MMTVels positive saliva demonstrated a cytoplasmic positivity in rare cells with morphology compatible with exfoliated epithelial cells (Figure 2). The p53 probe used as internal control hybridized inside the nucleus, as expected. The MMTV probe used is too short to reveal possible genome integration sites in the nucleus. On the other hand, the fact that it can bind also RNA well explains the presence of cytoplasmic signals.

### MMTV sequences are present in human salivary glands with a frequency similar to that of the saliva of the adult general population

Thirty-seven formalin fixed and paraffin embedded blocks (FFPE) of normal salivary gland tissue, from the same number of individuals, were obtained from the files of the Department of Pathology of the Pisa University Hospital and of the “San Rossore” Hospital in Pisa.

MMTVels DNA was present in 8.1% of cases (3/37), with 5% of females (1/20) and 11.7% of males (2/17) (Table 1). As said before, the saliva of the adult general population is positive for MMTV in 10.6% of cases.

### Salivary glands contain the MMTV-p14 protein

**Figure 3:**
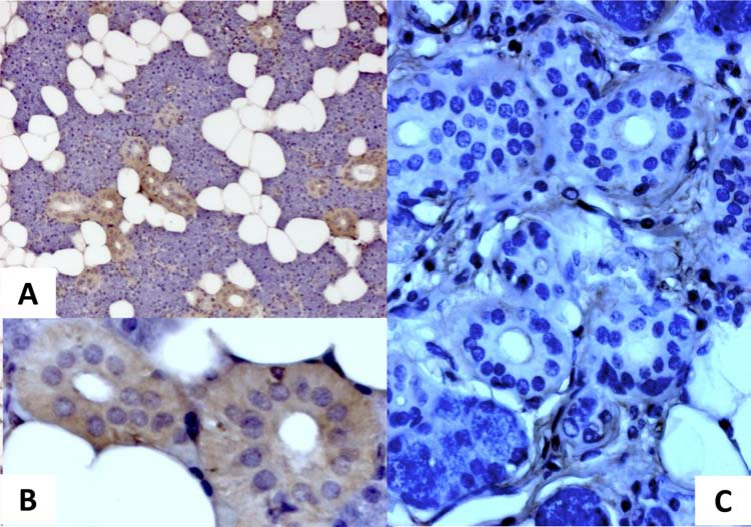
Immunohistochemical analysis of p14 protein expression in human salivary glands **A.** a cytoplasmic positivity for p14 is well evident in a sample positive for MMTVels. **B.** higher magnification of A; **C.** absence of p14 in a sample negative for MMTVels.

MMTV-p14 is the signal peptide of the MMTV envelope precursor, localized in the nucleolus of cells harboring the virus and with nucleo-cytoplasmic shuttling [15]. p14 expression was analyzed by immunohistochemistry in the two groups of salivary glands resulted positive and negative for MMTVels DNA respectively. All three MMTVels positive glands showed a cytoplasmic positivity for p14, whereas MMTVels negative cases resulted negative (Figure 3). In a few nucleoli of the positive samples a faint staining was also observed.

### Saliva samples are not contaminated by mouse DNA

**Figure 4:**
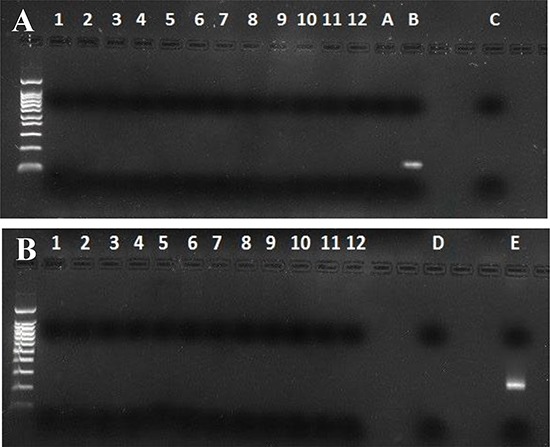
Murine mitochondrial DNA and Intracisternal A Particles long terminal repeats (IAP LTRs) PCR in human saliva **A)** mtDNA: MMTVels positive saliva samples (lanes 1–12); **A** and **C.** are PCR and nested PCR negative controls; **B.** is mouse DNA. All human samples result negative. **B)** IAP LTRs: MMTVels positive saliva samples (lanes 1–12); **D.** is a PCR negative control; **E.** is mouse DNA. All human samples result negative.

A possible mouse DNA contamination of MMTVels positive saliva samples was excluded by performing murine mitochondrial DNA (mtDNA) and Intracisternal A Particle (IAP) LTRs PCR. All tested samples resulted free of mouse DNA (Figure 4).

## DISCUSSION

In mouse, MMTV is transmitted from the mother to the offspring by milk [2]. Milk contains a high quantity of viral particles [16], most of which are destroyed by gastric juice [17]. In the ileum, at the Peyer's patches, particles enter into the newborn-type epithelial cells and M cells by a process of endocytosis [18]. The interaction between MMTV Env protein and Toll-like receptor 4 allows the infection of B cells, that present Sag (Super Antigen) to CD4^+^ T cells. These activated T cells stimulate and recruit other B and T cells, with the result of a further activation of immune cells and of the amplification of a reservoir of MMTV infected cells. Finally, B cells reach the mammary gland, where they are able to spread the infection to epithelial cells. These data [14, 19] make clear that, differently from other types of viruses, in the case of MMTV the presence of lymphoid structures at the entrance site and a working immune system are indispensable prerequisites for infection.

In mouse, MMTV is the causative agent of mammary tumors. Moreover, this experimental model was of great relevance for the understanding of many of the biological and morphological characteristics of human breast cancer [4].

Notwithstanding the strong similarities between mouse and human tumors, the efforts to demonstrate a viral origin for HSBC were unsuccessful. However, nowadays there is a substantial amount of data supporting this possibility, such as: the presence of MMTV sequences in a percentage between 30% and 40% of infiltrating HSBC [5, 6]; the presence of MMTV sequences shown by CISH in the nucleus of tumor cells [10]; the demonstration of a strict relationship between the presence of MMTV sequence and the progression of HSBC (normal gland, atypical ductal epithelial hyperplasia, ductal carcinoma in situ) [10]; the production of viral particles in primary cultures of human breast cancer [7]; the ability of MMTV to infect human cells in culture [8, 20]; the identification of a molecular mechanism that facilitates entrance of MMTV in non-murine cells [21].

The viral hypothesis for HSBC would receive a strong support if a route of infection could be defined for humans. Unfortunately, there are biological reasons that make milk improbable as vehicle in MMTV transmission in humans. In fact, in mouse a critical number of intact viral particles is necessary for the infection and this is obtained by a long period of breastfeeding, in that at any lactation the largest part of virus is destroyed in the stomach [17, 18, 22]. On the other hand, viral particles have been demonstrated in human milk [23], but in a very low concentration [24], and human milk has been shown to exert a destructive action on MMTV particles [24, 25]. Moreover, since a long time in humans lactation is considered protective against breast cancer or ineffective [26]. However, the presence of traces of MMTV in human milk, in terms of viral particles and sequences [11, 12], well supports the possibility of a human MTV.

Very few papers analyzed, unsuccessfully, the possibility of a zoonotic MMTV infection of humans, directly from mouse or by an intermediate host, such as a dog or a cat [27–30].

Abandoning the idea of human milk as a route for MMTV transmission, we went back once more to the mouse model, where we found that MMTV particles are produced in salivary glands too [14]. Moreover, saliva is a very common route of inter-human transmission for viral infections.

Our results (Tables 1) demonstrate that MMTV DNA is present in saliva and in salivary glands of healthy adults in a very similar percentage (10.6% and 8.1% respectively). At the same time, the percentage of positive cases in pediatric age is almost three times higher; these data are not surprising, considering that kids are much more prone than adults to infections, mainly viral. Very interestingly, positive cases go up to almost 60% in HSBC patients. Discrepancies in percentage of positivity found for MMTV DNA and RNA and for the combination of both can be consequence of a different MMTV status, in active replication or in latency.

Finally, the absence of MMTVels in the saliva of newborns eliminates the possibility of a trans-placental route and strongly supports the possibility of an inter-human transmission of MMTV.

This study for the first time reports a whole transcriptome analysis of MMTV sequences in a human sample, showing a much higher presence of RNA MMTV reads in the saliva of a breast cancer patient compared to tissues obtained from non-cancer breasts used as negative controls (Fig. 1).

As molecular morphological approach, we used FISH analysis on saliva resulted positive for both DNA and RNA MMTVels by fluorescent nested PCR (Fig. 2). Few clear signals are evident in the cytoplasm of cells that morphologically look like epithelial exfoliated cells. The probe used is capable of hybridizing also RNA sequences, suggesting the presence of viral particles in the cytoplasm. This approach was used only once in the past, showing signals in different chromosomes in primary cultures of metastatic breast cancer cell lines established from pleural effusions of patients [31].

Finally, the immunohistochemical analysis of salivary gland tissue showed, according to previous data [15], a clear cytoplasmic positivity for the MMTV protein p14 in PCR positive cases, whereas the absence of the same protein was documented in PCR negative cases.

An experimental paper at the end of 90′ [32] demonstrates that adult mice can be infected by MMTV through nasal inoculation of infected milk; in fact, viral particles are able to cross the respiratory epithelium and to initiate their infectious cycle in the lymphoid tissue associated to nasal mucosa (NALT). This paper shows two highly relevant facts: 1) MMTV can infect adult animals, 2) the site of entrance of MMTV can be different from the small intestine, provided the presence of a mucosa and of a mucosa associated lymphoid tissue, where the virus can be amplified before spreading to exocrine glands. This report strongly supports our finding, suggesting that in humans MMTV, spread by saliva, can reach the nasopharyngeal mucosa associated lymphoid tissue and the lymphatic structures of the Waldeyer's ring, where it can be amplified and from where it can spread to other sites, breast included.

The available data also suggests that the presence of MMTV in humans is not consequence of a zoonosis, but of a cross-species transmission taken place in ancient times, with a consequent inter-human transmission by saliva. A similar event has already been demonstrated for HIV, from chimpanzees to humans [33].

This hypothesis is well supported by a very recent paper [34] reporting a frequent presence of human beta retrovirus (HBRV) infection at the site of disease in patients with primary biliary cirrhosis and in biliary epithelium of patients with autoimmune hepatitis and cryptogenic liver disease. HBRV is a new name that authors give to MMTV.

Finally, in recent years data about the role of XMRV in human prostate cancer were questioned on the basis of a possible contamination by murine material. This occurrence was excluded in our study by showing the absence of mouse mitochondrial DNA and of intracisternal A particle LTR, as suggested [35].

This paper strongly supports a viral etiology for HSBC offering a possible route of infection. However, additional studies are necessary to demonstrate the molecular mechanism of the process. If this hypothesis is proven, the possibility for human breast cancer of a primary prevention strategy by vaccines would be possible.

## MATERIALS AND METHODS

### Salivary glands

Thirty-seven formalin fixed and paraffin embedded blocks (FFPE) of normal salivary gland tissue, from the same number of individuals, were obtained from the files of the Department of Pathology of the Pisa University Hospital and of the “San Rossore” private Hospital in Pisa. The patients were 17 males and 20 females, with a mean age of 54 years (with the exception of a 6 months old girl). Parotid was resected in 35 cases and a minor salivary gland in 2 cases. Surgery was due to a benign primary tumor of salivary glands in 19 cases, to a malignant primary tumor of salivary glands in 2 cases, to a non neoplastic lesion of the salivary glands in 8 cases, to a malignant tumor of the contiguous tissues (skin, subcutaneous tissue, lymph nodes) in 4 cases, to a benign pathology of the contiguous tissues in 3 cases, to a thrombosis of a intra-parotid blood vessel in one case. All blocks were made anonymous prior to the laboratory procedures, according to the rules of the Ethics Committee of the Pisa University Hospital.

### Laser Microdissection

Salivary gland tissues were laser microdissected (Leica Microsystems, Wetzlar, Germany). Sections 2 μm thick were cut from each case using a new microtome blade for each slide, obtaining a total of 10,000 to 15,000 cells. Stromal and inflammatory cells were carefully excluded.

### Saliva

Saliva samples were collected anonymously at the Pisa University Hospital from a total of 235 consecutive individuals, grouped as follows: 56 breast cancer female patients, 132 healthy adult blood donors of both genders, 30 pediatric patients of both genders, 17 newborns of both genders. In newborns oral swabs were used (DNA Genotex, Ontario, Canada). For all others subjects, 3 ml of saliva were collected in 50ml empty tubes and treated within 2 hours for DNA and RNA isolation.

### DNA isolation

*Saliva samples*: DNA isolation was performed using Genomic DNA from Tissue kit (Macherey-Nagel, Duren, DE), following the manufacturer's protocol at the section “DNA extraction from clinical samples”.

*Salivary tissues*: samples were incubated overnight at 37°C in a home-made lysis buffer containing 10 mM Tris-HCl, 1 mM ethylenediamine tetraacetic acid (EDTA), 1% Tween 20, and 0.1 mg/ml proteinase K (Qiagen, Venlo, Netherlands). Afterwards the samples were processed for DNA amplification.

### RNA isolation and RT-PCR

RNA extraction from saliva samples was performed using the QIAamp viral RNA mini kit (Qiagen, Venlo, Netherlands) according to manufacturer's protocol. RNA was reverse transcribed in a final volume of 20 μl, using the kit from Invitrogen.

### MMTVels detection: PCR

Fluorescence-nested PCR was used to detect the presence of the DNA and RNA MMTV *env*-like sequence. The pairs of primer were designed on the basis of the sequence available in GenBank (accession number AF243039). The protocol used has previously been described [10].

### Research of mouse DNA

The presence of contaminating mouse DNA was analyzed by performing murine mitochondrial DNA and IAP LTRs PCR, according to Robinson et al [35].

### FISH analysis

Two ml of a saliva sample positive at MMTVels PCR detection was collected in a ThinPrep PreservCyt Solution (Hologic, Inc., Bedford, MA, USA), and plated on glass slides as a monolayer using the ThinPrep 2000 System (CYTYC Corporation Marlborough, MA, USA).

The HMTV FISH probe was prepared in-house by amplification of a 2.7-kb PCR product using primers previously described [31]. FISH experiments were performed using the Cytology FISH kit (Dako, Glostrup, Denmark).

### Immunohistochemistry

Immunohistochemical assay was performed on 5 μm-thick paraffin sections. The antigen retrieval was achieved with MS-unmasker solution (DIAPATH, Martinengo, BG, Italy) in microwave. Histostain–Plus kit (Invitrogen, Carlsbad, CA, USA) was used according to manufacturer's protocol.

The slides were incubated with a primary antibody, rabbit polyclonal anti-MMTV-p14 (1:500 dilution), then developed with diaminobenzidine chromogen (DAB) (DAKO, Glostrup, DK) and counterstained with hematoxylin. Negative control included the omission of the primary antibody.

### Whole transcriptome analysis (RNA-seq)

#### Library and template preparation

RNA-seq was performed using Ion Proton™ Sequencer (Ion Torrent, Life Technologies, Grand Island, NY). The starting material of 500 ng of total RNA was treated with the Low Input RiboMinus Eukaryote Kit v2 (Ambion, Life Technologies, Grand Island, NY) to remove rRNA species. The manufacturer's protocols were followed for cDNA and template production. 165 millions wells Proton™ I Chip were used for sequencing.

#### Mapping of RNA-Seq reads using TopHat 2 and Bowtie

Bioinformatics analysis was carried out using several command line software included in Bio-Linux (http://nebc.nerc.ac.uk/tools/bio-linux/bio-linux-7-info). Using TopHat2 the reads, previously filtered for the quality and length, were processed and aligned to a reference sequence obtained by concatenating the H. sapiens reference genome (build hg19) and MMTV virus sequence (NC_001503), using as reference annotation a custom gtf annotation file. This file was built by joining the Genomes UCSC hg19 annotation file and MMTV annotations. The unmapped reads, generated from the first step, were re-aligned by using Bowtie2. The reads mapped with Tophat2 and Bowtie2 were then merged by using the Picard command SamMerge.

#### Transcript assembly with Cufflinks

the merged reads were processed with Cufflinks. Cufflinks normalizes the RNA-Seq fragment counts to estimate the abundance of each transcript. Abundance was measured in the units of fragments per kilobase of exon per million fragments mapped (FPKM). For this analysis the same GTF annotation file used for Tophat2 was used to guide the assembly. The graphical representation of expression value was generated by using RNASeqViewer V. 0.8.0.

## Abbreviations

BRCA: breast cancer gene
CD4: cluster of differentiation 4 protein
CISH: chromogenic in situ hybridization
DCIS: ductal carcinoma in situ
DNA: deoxyribonucleic acid
env: MMTV envelope gene
FFPE: formalin fixed and paraffin embedded tissues
FISH: fluorescent in situ hydridization
FPKM: fragments per kilobase of exon per million fragments mapped
HBRV: human beta retrovirus
HIV: human immunodeficiency virus
HPV: human papilloma virus
HSBC: human sporadic breast carcinoma
IAP: intracisternal A particle
LTR: long terminal repeats
MMTV: mouse mammary tumor virus
MMTVels: MMTV env gene–like exogenous sequence
MMTV-p14: MMTV signal peptide
mtDNA: mitochondrial DNA
p53: p53 gene
NALT: nasal associated lymphoid tissue
PCR: polymerase chain reaction
RNA: ribonucleic acid
Sag: super antigen
XMRV: xenotropic murine leukemia virus-related virus
